# The Effects of Surgical Approaches and Enhanced Recovery Protocols on the Cost Effectiveness of Radical Cystectomy

**DOI:** 10.3390/jpm12091433

**Published:** 2022-08-31

**Authors:** Eyal Kord, Moshe Leshno, Miki Haifler

**Affiliations:** 1 Section of Urology and Renal Transplantation, Virginia Mason Hospital and Seattle Medical Center, Seattle, WA 98101, USA; 2 Coller School of Management, Tel Aviv University, Tel Aviv 6997801, Israel; 3 Department of Epidemiology and Preventive Medicine, Tel Aviv University, Tel Aviv 6997801, Israel; 4 Department of Urology, Sheba Medical Center, Affiliated to the Sackler Faculty of Medicine, Tel-Aviv University, Tel-Aviv 52621, Israel

**Keywords:** cost effectiveness, radical cystectomy, robotic surgery, enhanced recovery protocol, urinary diversion

## Abstract

Enhanced recovery protocols and robotic approaches to radical cystectomy are known to reduce perioperative complications; however, the most cost-effective strategy is unknown. We aim to assess the cost effectiveness of radical cystectomy with different surgical techniques and perioperative treatment protocols. We performed a meta-analysis of studies comparing open radical cystectomy (ORC), robotic assisted radical cystectomy (RARC) using extracorporeal (ECUD) or intracorporeal urinary diversion (ICUD) and enhanced recovery after surgery (ERAS) protocols. Operative time, transfusion, complication, Ileus, length of stay and re-admission rates were extracted. US costs for surgery, treatment, hospitalization and complications were obtained from the literature. Israeli costs were obtained from hospital administrative data. Two cost effectiveness models (US and Israel) were developed. The two most cost-effective strategies in both models were ORC with ERAS and RARC with ICUD and ERAS. RARC with ERAS produced the two most effective strategies with ICUD being dominant over ECUD. All strategies implementing the ERAS protocol were more effective than their parallel non-ERAS strategies. RARC with ICUD and ERAS is cost effective compared to ORC. ERAS protocol improves treatment effectiveness and lowers overall costs. ICUD was shown to be more effective and less costly in comparison to ECUD.

## 1. Introduction

Bladder cancer has the seventh highest incidence of all malignancies in the United States, with an estimated 81,400 new cases and 17,980 deaths in 2020 [1]. A total of 20 to 30% of patients will present with muscle-invasive bladder cancer (MIBC) at the time of initial presentation [2]. Radical cystectomy (RC), which is the gold standard therapy for localized MIBC, provides excellent local control; however, it is highly morbid, with lengthy hospital stays and a high complication rate [3]. Although considered the gold standard, the open approach for radical cystectomy (ORC) has been challenged in the past years by the minimally invasive robotic assisted approach (RARC). Several recent randomized control trials (RCT) and large retrospective studies comparing ORC and RARC have demonstrated that oncological outcomes were non-inferior in the robotic approach [4,5,6,7,8,9,10]. Urinary diversion after RARC can be performed with an open approach (extracorporeal method (ECUD)) or minimally invasively approach (intracorporeal urinary diversion (ICUD)). Several benefits to using an ICUD such as a decreased operative time and decreased risk of fluid imbalance have been suggested at the cost of a higher rate of complications and a prolonged learning curve [5].

Enhanced recovery after surgery (ERAS) pathways comprising early mobilization, feeding, neuromodulators and bowel activity promoting drugs have also been implemented and shown to shorten hospitalization and increase patient’s recovery [11]. Assessments of the treatment costs and effectiveness of RARC has been published in several studies in the past; however, most studies are based on single institution cohorts and do not take into account the evolution of RC with regard to ICUD and ERAS protocols.

Although RARC has been adopted in the US and Europe during the past several years, questions regarding the clinical benefits and the procedure’s value for money are still in debate. As cost effectiveness is impacted differently in different health care systems, we designed our experiment to examine our models in two different health care systems, the US system and the Israeli public health care system.

The aim of our study is to perform a cost effectiveness analysis (CEA) of RC including ORC and RARC with different methods of urinary diversion and implementations of ERAS protocols in two different healthcare systems.

## 2. Materials and Methods

### 2.1. Model Structure

**Figure 1:**
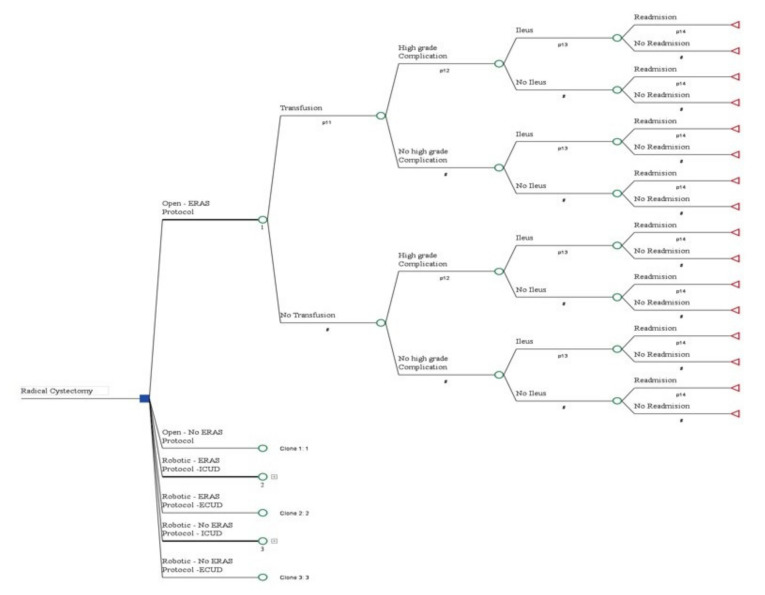
The Analytical Tree Model used in our analysis. Each node relates to a decision regarding patient care (ERAS, transfusion, complications, ileus and re-admission).

A decision analytical model (Figure 1) for the cost effectiveness (C/E) of RC was constructed and comprised the following 6 treatment strategies: 1) ORC without ERAS protocol; 2) ORC with ERAS protocol; 3) RARC with ECUD and without ERAS protocol; 4) RARC with ECUD and with ERAS protocol; 5) RARC with ICUD and without ERAS protocol; and 6) RARC with ICUD and with ERAS protocol. We constructed 2 models according to US and Israeli health care systems.

### 2.2. Effectiveness Metrics

The following effectiveness metrics were included in the model: 90 days high-grade complication rate (Clavien-Dindo 3–5), prolonged post-operative ileus, need for blood transfusion, 90 days re-admission rate, operative time (OT) and length of stay (LOS).

We performed a literature review based on the methods section of the PRISMA statement for systematic reviews. Our review process, PRISMA checklist, search strategy terms and flow diagram for the selected studies are described in Supplementary S1. We used the PUBMED and EMBASE electronic data sources in order to assess the point and range of the probability estimates for the different clinical events. Studies published between January 2010 and April 2021 were selected and screened for their relevance for this study. We included RCT’s, multicenter cohort studies and large retrospective studies comparing ORC vs. RARC, ICUD vs. ECUD or the use of ERAS in RC. Non-comparative studies, case reports and descriptions of surgical techniques were excluded. We selected the first 90 days after surgery as the studied time frame since most surgery-related adverse events occur within this time period. Variations in the rate of events between different studies were accounted for by performing a meta-analysis using the “Comprehensive meta-analysis” V2 (CMA) software. (Biostat Inc., Englewood NJ, USA).

### 2.3. Cost Estimates

The costs of surgery and events in the US model were derived from the literature review. The costs for the Israeli model were received from hospital administration (Shamir medical center) in New Israeli Shekels (NIS) and were converted to the United States Dollar (USD) using a 3.4 NIS/USD exchange rate relevant to February 2021 according to the International monetary fund reports. The cost data used in the decision analytic model are presented in Table 1. The mean values were used for the costs in the analysis. The hospital costs included fixed (robot acquisition, maintenance and insurance, disposable equipment, room and board), variable (operating room fee, medications) and professional (surgeons and anesthesia) costs. In addition, the costs of hospital stays, ERAS protocol and adverse events such as complications and re-admission were used to evaluate the overall cost of a specific treatment strategy. The costs were inflated to the date of the last surgery in the study. Inflation was applied using the consumer price index for medical costs in US dollars and NIS. The costs were assigned to patient events on the basis of the mean differences for a patient experiencing an event compared to patients not experiencing an event.

### 2.4. Preference Weights 

Preference weights (utilities) were obtained from literature reports on health utilities for clinical events (Supplementary S2). Preference weights were assigned on a scale from 0 (death) to 1.0 (perfect health).

### 2.5. Model Output

The primary output of the models is quality adjusted life years (QALY) calculated as follows:

QALY=∑PW×Ti

QALY—Quality Adjusted Life Years, PW—preference weight, Ti—time in health state i.

QALY together with costs were used to calculate the incremental C/E ratio (ICER) between each two strategies:

$$\mathrm{ICER}=\frac{\Delta \mathrm{Cos}t}{\Delta \mathrm{QALY}}=\frac{{\mathrm{Cos}t}_{\mathrm{str}1}-{\mathrm{Cos}t}_{\mathrm{str}2}}{{\mathrm{QALY}}_{\mathrm{str}1}-{\mathrm{QALY}}_{\mathrm{str}2}}$$

Str—strategy, QALY—quality adjusted life years.

The CEA was performed with TreeAge Pro v.2017 software (Eclipse Foundation, Williamstown, MA, USA). All methods for the CEA were modeled after previously described methods [22].

### 2.6. Sensitivity Analysis

A sensitivity analysis was performed in order to address the variability of the data collected from different studies. Top influencers on the C/E were determined using a tornado analysis. When variations were not available in the literature, a 15% variation was applied. In order to address the model’s uncertainty, a probabilistic sensitivity analysis was performed comparing the two leading strategies for both the US and the Israeli model. We used a Monte Carlo simulation of 100 trials and each trial included 10,000 individuals.

## 3. Results

The literature review yielded 28 articles comparing the different methods for RC. The complete meta-analysis outcomes are detailed in Supplementary S3.

The utilization of RARC decreased rates of blood transfusions, high-grade complications and ileus events while increasing the 90-day re-admission rate in comparison to the open approach. The implementation of the ERAS protocol, decreased hospital length of stay, rates of ileus and complications in comparison to each parallel strategy.

### 3.1. United States Model

**Figure 2:**
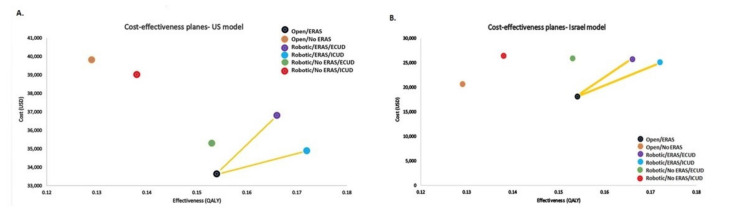
Cost effectiveness planes for the (**A**) US Model and (**B**) Israeli Model. While the costs in the two models are different, the dominant strategies are identical. The slopes of the yellow lines represent the ICER. In both models, ORC/ERAS and RARC/ICUD/ERAS are the most cost-effective strategies.

The combination of the RARC and ERAS protocol produced the two most effective strategies compared with ORC with ERAS. RARC/ICUD was dominant over RARC/ECUD. The C/E planes of the different strategies are depicted in Figure 2.

The performance of ICUD/ERAS was more effective than ECUD/ERAS and less expensive by USD 1919 (Table 1). Although ERAS added an average cost of USD 4550 per case, it reduced the overall cost of the calculated strategies in the open and ICUD arms by USD 4926 and USD 4128, respectively. This reduction in cost can be attributed to the shorter length of stay and fewer high-grade complications seen with ERAS.

The three leading strategies after comparing the effectiveness and costs were ORC/ERAS, RARC/ICUD/ERAS and RARC/ECUD/ERAS. All other strategies were more costly or less effective. The ICER between RARC/ICUD/ERAS vs. ORC/ERAS was USD 69,944 per QALY. In comparison, the ICER between RARC/ECUD/ERAS vs. ORC/ERAS was USD 264,833 per QALY.

**Figure 3:**
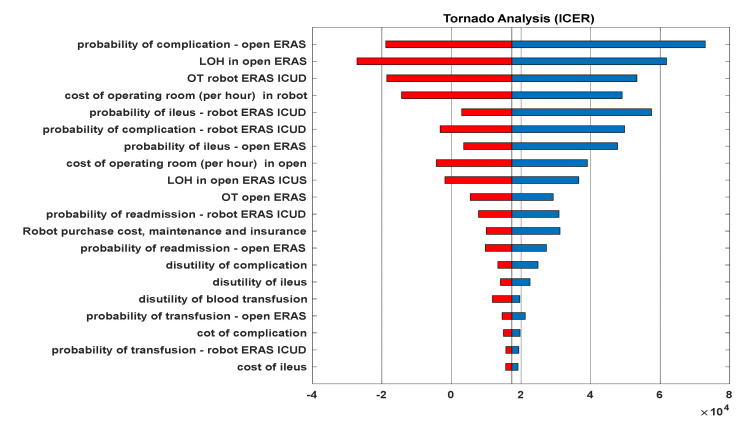
Tornado Plot of the US Model. The two most influential factors on CE are complication rate and length of stay in the open/ERAS strategy.

A sensitivity analysis using the Tornado plot (Figure 3) was performed to determine which variables had the most influence on the ICER between ORC/ERAS and RARC/ICUD/ERAS. The probability of the complications and the length of the hospital stay in ORC/ERAS were found to be most influential. A probabilistic sensitivity analysis comparing the two leading strategies (ORC/ERAS and RARC/ICUD/ERAS) and determining the boundaries of willingness to pay is presented in the Supplementary Material.

### 3.2. Israel Costs Model

In accordance with the US model, the three leading strategies in the Israeli model were ORC/ERAS (least costly), RARC/ICUD/ERAS (most effective) and RARC/ECUD/ERAS.

Similarly, ICUD/ERAS dominated ECUD/ERAS being more effective and less expensive by USD 564. All four robotic strategies were more costly than ORC.

ERAS lowered treatment costs and increased effectiveness in comparison to each parallel strategy. The difference between the most expensive (RARC/ICUD/ERAS) and inexpensive strategy (ORC/ERAS) was USD 8334, representing a 31.4% decrease in overall costs (Table 2).

When comparing the three leading strategies, RARC/ICUD/ERAS was more effective than ORC/ERAS and more costly by USD 7055, translating to an ICER of USD 391,944 per QALY. RARC/ECUD/ERAS was more effective than ORC/ERAS and more costly by USD 7619, translating to an ICER of USD 634,916 per QALY.

**Figure 4:**
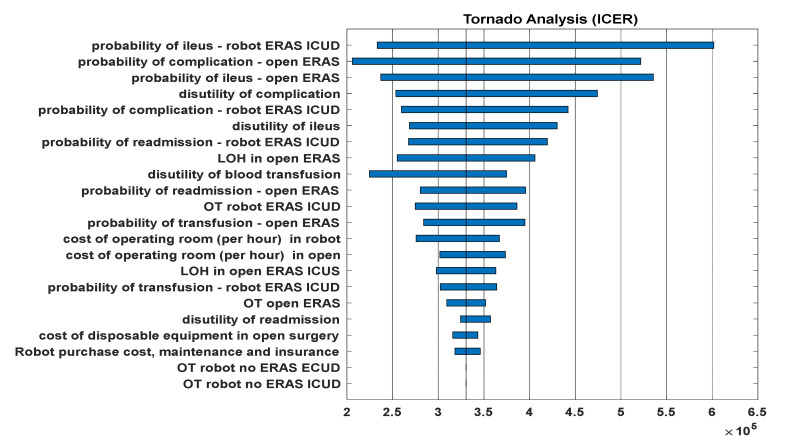
Tornado plot of the Israeli model. The three most influential factors on CE are ileus rate in the RARC/ERAS/ICUD and open/ERAS strategies and complication rate in the open/ERAS strategy.

The sensitivity analysis determined that the two variables influencing ICER the most were the probability of ileus in the RARC/ICUD/ERAS strategy and high-grade complications in the ORC/ERAS approach (Figure 4).

## 4. Discussion

The aim of our study was to examine the C/E of RC using different surgical approaches, urinary diversion methods and the use of the ERAS protocol while identifying the factors most influential on C/E.

Although there is no commonly agreed upon value or method for determining C/E thresholds, in the American health economic literature, a value of USD 50,000–USD 100,000 per QALY is commonly referenced. Our study demonstrates that in comparison to the standard ORC, RARC is cost effective under certain conditions. In the US model, the dominant robotic strategy was a combination of ICUD and ERAS. This strategy produced an ICER of 69,944 USD/QALY in comparison to the leading ORC/ERAS strategy. The second leading robotic strategy, ECUD and ERAS, produced and ICER of 264,833 USD/QALY. Although both leading robotic strategies were more costly than ORC/ERAS, they were also more effective as a result of the decreased rate of high-grade complications and ileus. The Israeli model produced similar results regarding the leading treatment strategies; however, both leading robotic strategies were not deemed cost effective in comparison to ORC/ERAS.

With regard to the factors influencing C/E, our sensitivity analysis showed that the change in the probability of complications and the length of hospital stays in the ORC/ERAS approach, and the operating time, probabilities of ileus and complications in the RARC/ICUD/ERAS approach, were the most influential factors on the ICER. These factors are in line with previous CEA studies performed on RC [12,19]. These factors could be considered modifiable factors in contrast to factors such as operating room, hospital board and disposable equipment costs. Although our analysis showed cost effectivity, measures such as performing RARC by fellowship-trained surgeons in high volume referral centres and increasing the number of robotic procedures per year may improve cystectomy outcomes and potentially decrease operative time, complications, the length of stays and overall costs [23].

Our findings support previous C/E studies examining RARC in comparison to ORC [12,19,20]. Hu et al. [20] utilized the SEER database and Medicare registry and performed propensity score matching between RARC and ORC patients, examining outcomes and costs. There was no difference in perioperative mortality, major complications, iatrogenic complications, and transfusions by surgical approach. Oncological outcomes in the two groups were similar. Similar to our study, a costs-of-care analysis demonstrated higher median inpatient costs for RARC (USD 24,051 [IQR USD 15,332–USD 32,078]) vs. ORC (USD 21,637 [IQR USD 12,567–USD 32,460], *p* = 0.08).

With regard to the urinary diversion method during RARC, i.e., ECUD vs. ICUD, our study showed an overall improved effectiveness and cost saving of USD 1919 using ICUD/ERAS vs. ECUD/ERAS. The dominance of combining robotic surgery with ERAS over robotic surgery alone has been shown in previous studies and has been attributed to a synergy of minimally invasive surgery with other components of the ERAS protocol [24]. In an RCT by Bochner et al. [4], comparing 60 patients who underwent RARC with ECUD and ERAS, and 58 patients who underwent ORC with ERAS, a cost comparison was performed. Their results showed that RARC was associated with increased costs in comparison to ORC. Although they did not perform a cost effectiveness analysis, since the only advantage for RARC in that trial was less blood loss, RARC with ECUD and ERAS would probably be deemed not cost effective, similar to the results of our study.

The advantages of ICUD over ECUD is a matter of controversy and is under examination in current ongoing randomized controlled trials. The largest reports of ICUD outcomes were published in two large retrospective studies [5,6] that demonstrated that ICUD patients had more overall complications (66% vs. 58%, *p* = 0.01) and re-admissions (27% vs. 17%, *p* = 0.01). However, the study results showed that ICUD is feasible with advantages such as less blood transfusions and shorter operative times, probably due to the conversion to open urinary diversion with ECUD.

In a recent RCT by Maibom et al. [25] examining RARC, 25 patients undergoing RARC with ICUD and ERAS were compared to 25 patients undergoing ORC with ERAS. The main outcome of this small feasibility study was the possibility to perform a double-blind study in the setting of robotic vs. open surgery. The results of the secondary outcomes of this study were in line with past RCT’s examining ECUD, showing decreased blood loss and increased operative time in the robotic arm. The high-grade complication rates were similar in both arms (12% for RARC and 8% for ORC) and were relatively low in comparison to previous trials. The length of stay and the use of analgesics were not significantly different.

In our study, the implementation of an ERAS protocol improved treatment effectiveness while reducing costs in all strategies. These conclusions are in line with recent literature describing an improvement in RC outcomes with the implementation of ERAS protocols [26].

When comparing outcomes between the US and Israeli model in our study, similar results arose regarding the leading strategies. Although the leading robotic strategy was found to be a C/E strategy in the US model, in the Israeli model, both leading robotic strategies were above the acceptable healthcare boundaries of willingness to pay. The lack of C/E of RARC in the Israeli model stems from the large difference in cost between robotic and open surgery. While in the US model the difference between RARC/ICUD/ERAS and ORC/ERAS was USD 1259, in the Israeli model it was USD 7055. These differences are mostly related to model variations in the costs of robotic disposable equipment, robotic system purchase, maintenance and insurance but also to the costs of re-admissions and the treatment of high-grade complications.

Recently, a CEA based on clinical, quality of life and cost data from 180 RARC and 168 ORC patients operated in 19 medical centres in the Dutch healthcare system was published [27]. In their analysis, their effectiveness assessment was based on complication rates and patient-reported quality of life questionnaires. In contrast to our findings, no difference in effectiveness between RARC and ORC was demonstrated. From a healthcare perspective, RARC was found to be more costly by 4125 euros than ORC. According to these findings, ORC dominated RARC when compared in the Dutch healthcare system. Although during a 1-year follow up, every 3 months approximately 40% of the data from the patient-reported questionnaires were missing, which may have skewed the effectiveness assessment, this study highlights the potential differences in cost and effectiveness parameters between countries and healthcare systems. Furthermore, our study aimed to assess the impact of ERAS and ICUD on cost effectivity, which has not been done before.

There are several potential limitations to our study. First, evaluating clinical event probabilities from the literature depends on the studies included in the analysis and, as such, is potentially subjected to selection and publication bias. As 23 of the 28 studies included in the meta-analysis were non-randomized trials, a potential for different comorbidity profiles between the groups exists, thus affecting outcomes. In order to overcome this limitation, we performed a meta-analysis, which also allowed us to avoid the bias of using only single institutional data. Furthermore, the use of the confidence interval range for the clinical outcomes and performance of a sensitivity analysis mitigates this bias. Second, the possibility of variation in the ERAS protocols used in the studies can potentially interfere with the impact and cost assessment of the ERAS arms in our study. However, the protocols used were mostly similar and the advantages in use of the ERAS protocols in the literature have been shown without consideration of any single specific item. As the use of Alvimopan was specified in only 4 out of the 18 studies which assessed ERAS, we decided not to include the cost of the drug in the total ERAS cost calculations. Thirdly, the cost variation of treatment and complications within different health systems may be significant. The sensitivity analysis and willingness to pay curves allow one to control for these issues.

## 5. Conclusions

RARC with ICUD and an ERAS protocol is cost effective in comparison to ORC in the US model. The implementation of ERAS protocols improved treatment effectiveness and lowered overall costs. ICUD was shown to be more effective and less costly in comparison to ECUD.

## Figures and Tables

**Table 1:** Study cost data.

Variable	Cost in US Model	US Model Costs References	Cost in Israeli Model
**ORC**			
Operating room—surgical and anesthesia (1 h)	USD 2020	[12,13,14]	3600 NIS (USD 1059)
Disposable equipment	calculated in operating room fee	[14,14]	NIS (USD 3509) 11,932
Hospitalization fee (per day)	USD 840	[12,13,14]	NIS (USD 747) 2538
Medication (per day)	USD 217	[15,16]	calculated in hospitalization fee
ERAS protocol	USD 440 (per day)	[16,17,18]	3500 NIS (USD 1029) (per hospitalization)
ERAS home therapy	USD 590	[16,17,18]	calculated in ERAS protocol
**RARC**			
Robot purchase, maintenance and insurance (per case)	USD 1299	[12,13,14,19]	10,882 NIS (USD 3200)
Operating room—surgical and anesthesia (1 h)	USD 2323	[12,13,14]	3600 NIS (USD 1059)
Disposable equipment	calculated in operating room fee	[14]	(USD 6928) NIS23,555
Hospitalization fee (per day)	USD 840	[12,13,14]	(USD 747) NIS2538
Medication (per day)	USD 217	[15,16]	calculated in hospitalization fee
ERAS protocol	USD 440 (per day)	[16,17,18]	3500 NIS (USD 1029) (per hospitalization)
ERAS home therapy	USD 590	[16,17,18]	calculated in ERAS protocol
**General**			
Blood transfusion (2 units)	USD 268	[12]	(USD 71) NIS240
High-grade complication	27,936USD	[3,20]	(USD 4357) NIS14,814
Re-admission	USD 4847	[3,20,21]	NIS (USD 2986)10,152
Prolonged ileus	10,246USD	[3,17,20]	(USD 1500) NIS5100

**Table 2:** Cost effectiveness of treatment strategies in US and Israel models.

Strategy		Cost (USD)	Effectiveness (QALY)	Compared to Strategy	ICER (USD)
**US Model**					
1	Open/ERAS	33,635	0.154		
2	Open/No ERAS	39,821	0.129	1	−247,440
3	Robotic/ERAS/ECUD	36,813	0.166	1	264,833
4	Robotic/No ERAS/ECUD	35,303	0.153	5	−21,526
5	Robotic/ERAS/ICUD	34,894	0.172	1	69,944
6	Robotic/No ERAS/ICUD	39,022	0.138	5	−121,411
**Israel Model**					
1	Open/ERAS	18,179	0.154		
2	Open/No ERAS	20,726	0.129	1	−101,880
3	Robotic/ERAS/ECUD	25,798	0.166	1	634,916
4	Robotic/No ERAS/ECUD	25,983	0.153	5	−39,421
5	Robotic/ERAS/ICUD	25,234	0.172	1	391,944
6	Robotic/No ERAS/ICUD	26,513	0.138	5	−37,617

## Data Availability

The datasets generated during and/or analyzed during the current study are available from the corresponding author upon reasonable request.

## Supplementary Materials

The following are available online at https://www.mdpi.com/article/10.3390/jpm12091433/s1, Supplementary S1: Literature review search strategy terms; Supplementary S2: Study Preference weights; Supplementary S3. Meta-analysis outcomes and forest plots.
