# Supplementary material for: The Effects of Surgical Approaches and Enhanced Recovery Protocols on the Cost Effectiveness of Radical Cystectomy
Source: J Pers Med. 2022 Aug 31;12(9):1433. doi: 10.3390/jpm12091433 (PMC9502617; doi:10.3390/jpm12091433)
Supplement: Supplementary file 1 [file jpm-12-01433-s001.zip › jpm-1872929-supplementary.pdf]

Supplementary material:

Supplementary S1. Literature review search strategy terms

The following search strategy terms were used for the PUBMED search: ((cystectomy[Title/Abstract]) OR (cystectomy[MeSH Terms])) AND ((robot*[Title/Abstract]) OR (recovery[Title/Abstract])) AND ("2010/01/01"[PDat] : "3000/12/31"[PDat]) OR ("2010/01/01"[PDat] : "3000/12/31"[PDat])).

For the EMBASE search, the following search strategy terms were used: Query('cystectomy'/exp OR cystectomy:ab,ti) AND (robot*:ab,ti OR 'robot assisted surgery'/exp OR recovery:ab,ti) AND (2010:py OR 2011:py OR 2012:py OR 2013:py OR 2014:py OR 2015:py OR 2016:py OR 2017:py OR 2018:py OR 2019:py OR 2020:py) AND 'article'/i

2020 PRISMA methods checklist:

Section and Topic	Item #	Checklist item	Location where item is reported
**METHODS**			
Eligibility criteria	5	Specify the inclusion and exclusion criteria for the review and how studies were grouped for the syntheses.	Methods
Information sources	6	Specify all databases, registers, websites, organisations, reference lists and other sources searched or consulted to identify studies. Specify the date when each source was last searched or consulted.	Methods
Search strategy	7	Present the full search strategies for all databases, registers and websites, including any filters and limits used.	Online resource 2
Selection process	8	Specify the methods used to decide whether a study met the inclusion criteria of the review, including how many reviewers screened each record and each report retrieved, whether they worked independently, and if applicable, details of automation tools used in the process.	Methods
Data collection process	9	Specify the methods used to collect data from reports, including how many reviewers collected data from each report, whether they worked independently, any processes for obtaining or confirming data from study investigators, and if applicable, details of automation tools used in the process.	Methods
Data items	10a	List and define all outcomes for which data were sought. Specify whether all results that were compatible with each outcome domain in each study were sought (e.g. for all measures, time points, analyses), and if not, the methods used to decide which results to collect.	Methods
	10b	List and define all other variables for which data were sought (e.g. participant and intervention characteristics, funding sources). Describe any assumptions made about any missing or unclear information.	Methods
Study risk of bias assessment	11	Specify the methods used to assess risk of bias in the included studies, including details of the tool(s) used, how many reviewers assessed each study and whether they worked independently, and if applicable, details of automation tools used in the process.	
Effect measures	12	Specify for each outcome the effect measure(s) (e.g. risk ratio, mean difference) used in the synthesis or presentation of results.	Online resource 4
Synthesis methods	13a	Describe the processes used to decide which studies were eligible for each synthesis (e.g. tabulating the study intervention characteristics and comparing against the planned groups for each synthesis (item #5)).	Online resource 2
	13b	Describe any methods required to prepare the data for presentation or synthesis, such as handling of missing summary statistics, or data conversions.	Methods
	13c	Describe any methods used to tabulate or visually display results of individual studies and syntheses.	Online resource 4
	13d	Describe any methods used to synthesize results and provide a rationale for the choice(s). If meta-analysis was performed, describe the model(s), method(s) to identify the presence and extent of statistical heterogeneity, and software package(s) used.	Methods
	13e	Describe any methods used to explore possible causes of heterogeneity among study results (e.g. subgroup analysis, meta-regression).	
	13f	Describe any sensitivity analyses conducted to assess robustness of the synthesized results.	Methods
Reporting bias assessment	14	Describe any methods used to assess risk of bias due to missing results in a synthesis (arising from reporting biases).	
Certainty assessment	15	Describe any methods used to assess certainty (or confidence) in the body of evidence for an outcome.	Methods

For more information, visit: http://www.prisma-statement.org/

CONSORT diagram for studies reviewed.

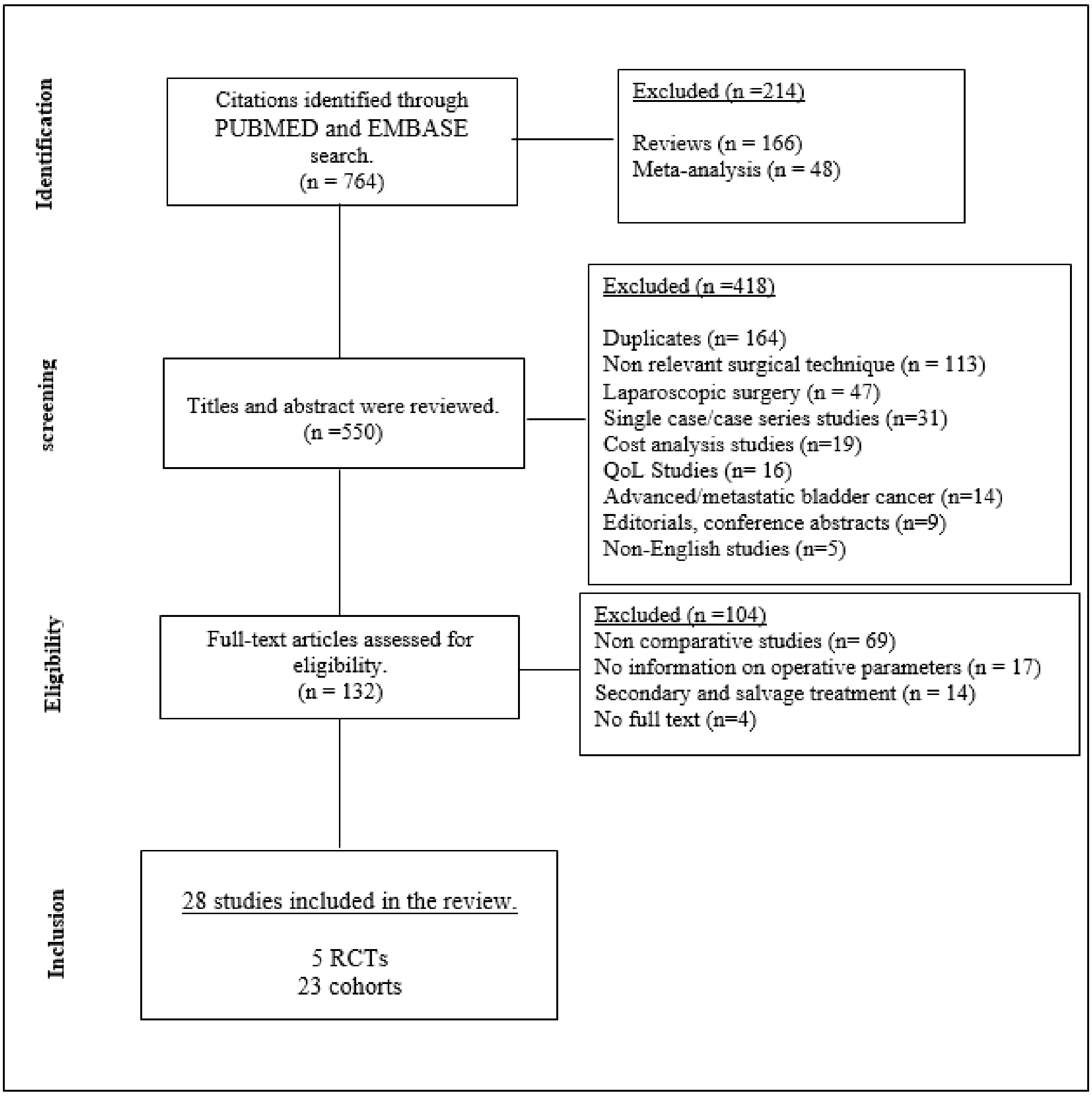

Flow Diagram for studies reviewed.

Supplementary S2. Study Preference weights.

Parameter	Value	Range	Reference
Utility of Radical cystectomy without adverse events	0.8	0.6-1	[1,2]
Utility of high grade complication	0.7	0.5-0.9	[1–3]
Utility of prolonged ileus	0.7	0.5-0.9	[1–3]
Utility of re-admission	0.9	0.7-0.95	[2,4,5]
Utility of blood transfusion	0.9	0.7-0.95	[6–8]

Supplementary S3. Meta-analysis outcomes and forest plots.

Meta-Analysis		
**ORC ERAS**	Mean	SD
Transfusion rate	0.321	0.060
High grade complications rate	0.266	0.042
Ileus rate	0.178	0.046
Readmission rate	0.181	0.048
**ORC No ERAS**		
Transfusion rate	0.377	0.049
High grade complications rate	0.440	0.097
Ileus rate	0.335	0.052
Readmission rate	0.128	0.023
**RARC ICUD ERAS**		
Transfusion rate	0.150	0.034
High grade complications rate	0.156	0.028
Ileus rate	0.094	0.054
Readmission rate	0.208	0.063
**RARC ECUD ERAS**		
Transfusion rate	0.173	0.038
High grade complications rate	0.182	0.031
Ileus rate	0.143	0.037
Readmission rate	0.206	0.036
**RARC ICUD no ERAS**		
Transfusion rate	0.050	0.007
High grade complications rate	0.261	0.043
Ileus rate	0.475	
Readmission rate	0.204	0.078
**RARC ECUD no ERAS**		
Transfusion rate	0.112	0.031
High grade complications rate	0.164	0.031
Ileus rate	0.281	0.125
Readmission rate	0.393	0.086

Hospital length of stay meta-analysis outcomes.

**Length of hospitalization(days)**	Median	LCI	UCI
Open ERAS	8.22	6.07	10.38
Open No ERAS	11.1	8.53	13.67
Robotic ECUD ERAS	8.54	6.66	10.43
Robotic ECUD No ERAS	8.77	5.73	11.81
Robotic ICUD ERAS	7.52	6.59	8.46
Robotic ICUD No ERAS	10.3	8.43	12.16

Operative time meta-analysis outcomes.

**Operative Time (hour)**	Median	LCI	UCI
Open ERAS	5.20	4.78	5.63
Open No ERAS	5.76	5.42	6.11
Robotic ECUD ERAS	6.26	5.54	6.97
Robotic ECUD No ERAS	6.60	5.89	7.31
Robotic ICUD ERAS	6.61	5.49	7.74
Robotic ICUD No ERAS	5.89	5.33	6.44

Meta Analysis

Study name	Rate	Standard error	Variance	Lower limit	Upper limit	Z-Value	p-Value
Parekh et al 2013	0.500	0.162	0.026	0.182	0.818	3.082	0.002
Prekh et al 2018	0.450	0.054	0.003	0.343	0.557	8.270	0.000
Daneshmand et al.	0.210	0.044	0.002	0.124	0.296	4.806	0.000
Koupparis et al.	0.410	0.086	0.007	0.242	0.578	4.792	0.000
Palumbo et al.	0.257	0.059	0.003	0.141	0.373	4.361	0.000
Chen et al.	0.409	0.034	0.001	0.342	0.476	11.879	0.000
Zhang et al.	0.130	0.027	0.001	0.078	0.182	4.904	0.000
	0.321	0.060	0.004	0.203	0.438	5.350	0.000

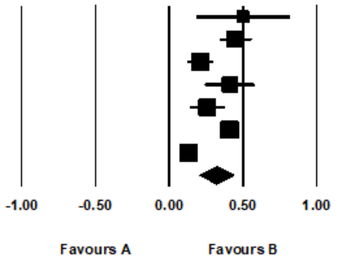

ORC ERAS – Transfusion rate.

Meta Analysis

Study name	Rate	Standard error	Variance	Lower limit	Upper limit	Z-Value	p-Value
Bochner et al	0.210	0.060	0.004	0.092	0.328	3.490	0.000
Khan et al	0.200	0.100	0.010	0.004	0.396	2.000	0.046
Nix et al	0.250	0.112	0.013	0.031	0.469	2.236	0.025
Parekh et al 2013	0.250	0.115	0.013	0.025	0.475	2.179	0.029
Parekh et al 2018	0.220	0.038	0.001	0.145	0.295	5.783	0.000
Daneshmand et al	0.420	0.062	0.004	0.299	0.541	6.797	0.000
Koupparis et al	0.474	0.092	0.008	0.294	0.654	5.152	0.000
Palumbo et al	0.531	0.085	0.007	0.365	0.697	6.268	0.000
Smith et al	0.120	0.067	0.004	-0.011	0.251	1.800	0.072
Djaladat et al	0.243	0.038	0.001	0.169	0.317	6.408	0.000
Chen et al	0.107	0.018	0.000	0.072	0.142	6.076	0.000
	0.266	0.042	0.002	0.184	0.348	6.345	0.000

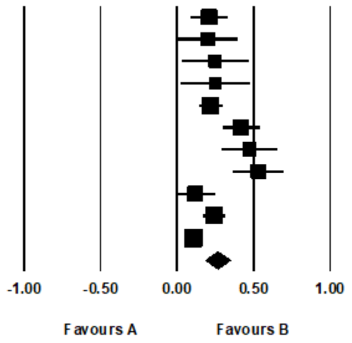

ORC ERAS – High grade complication rate.

Meta Analysis

Study name	Rate	Standard error	Variance	Lower limit	Upper limit	Z-Value	p-Value
Bochner et al	0.290	0.071	0.005	0.151	0.429	4.101	0.000
Khan et al	0.350	0.132	0.018	0.091	0.609	2.646	0.008
Nix et al	0.150	0.087	0.008	-0.020	0.320	1.732	0.083
Parekh et al 2018	0.200	0.036	0.001	0.129	0.271	5.514	0.000
Smith et al	0.040	0.038	0.001	-0.035	0.115	1.039	0.299
Persson et al	0.160	0.072	0.005	0.019	0.301	2.227	0.026
	0.178	0.046	0.002	0.088	0.267	3.894	0.000

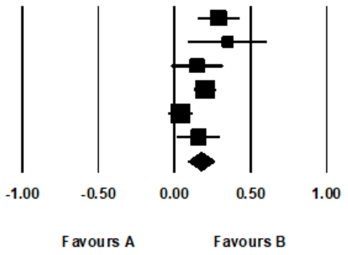

ORC ERAS – Ileus rate.

Meta Analysis

Study name	Rate	Standard error	Variance	Lower limit	Upper limit	Z-Value	p-Value
Modi et al 2019	0.270	0.008	0.000	0.254	0.286	33.137	0.000
Daneshmand et al 2014	0.210	0.044	0.002	0.124	0.296	4.806	0.000
Koupparis_et_al-2015	0.030	0.023	0.001	-0.015	0.075	1.296	0.195
Smith et al 2014	0.060	0.047	0.002	-0.032	0.152	1.273	0.203
Djaladat et al 2017	0.300	0.042	0.002	0.217	0.383	7.120	0.000
Chen et al 2019	0.320	0.030	0.001	0.260	0.380	10.507	0.000
Zhang et al 2020	0.080	0.021	0.000	0.039	0.121	3.847	0.000
	0.181	0.049	0.002	0.086	0.277	3.735	0.000

Statistics for each study

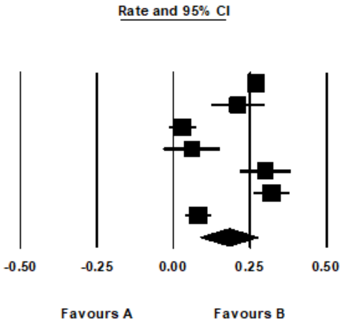

ORC ERAS – Re-admission rate.

Meta Analysis

Study name	Rate	Standard error	Variance	Lower limit	Upper limit	Z-Value	p-Value
Koupparis_et_al-2015	0.430	0.088	0.008	0.258	0.602	4.907	0.000
Palumbo et al 2018	0.400	0.100	0.010	0.204	0.596	4.000	0.000
Zhang et al 2020	0.279	0.033	0.001	0.215	0.343	8.484	0.000
Flamiatos et al 2019	0.500	0.058	0.003	0.386	0.614	8.631	0.000
Styn et al 2012	0.240	0.049	0.002	0.144	0.336	4.899	0.000
Kader et al 2013	0.470	0.069	0.005	0.336	0.604	6.856	0.000
	0.377	0.049	0.002	0.281	0.472	7.698	0.000

Statistics for each study

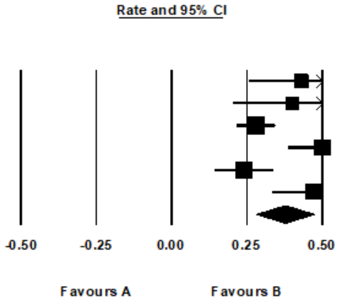

ORC no ERAS – Transfusion rate.

Meta Analysis

Study name	Rate	Standard error	Variance	Lower limit	Upper limit	Z-Value	p-Value
Tan et al 2018	0.302	0.082	0.007	0.141	0.463	3.686	0.000
Koupparis_et_al-2015	0.108	0.044	0.002	0.022	0.194	2.459	0.014
Palumbo et al 2018	0.225	0.075	0.006	0.078	0.372	3.000	0.003
Smith et al 2014	0.080	0.034	0.001	0.013	0.147	2.349	0.019
Persson et al 2015	0.510	0.114	0.013	0.286	0.734	4.460	0.000
Flamiatos et al 2019	0.570	0.062	0.004	0.449	0.691	9.216	0.000
Styn et al 2012	0.639	0.080	0.006	0.482	0.796	7.994	0.000
Ng et al 2010	0.924	0.094	0.009	0.739	1.109	9.803	0.000
Kader et al 2013	0.660	0.081	0.007	0.501	0.819	8.124	0.000
	0.440	0.097	0.009	0.249	0.631	4.520	0.000

Statistics for each study

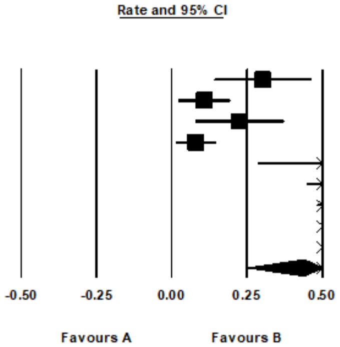

ORC no ERAS – High grade complication rate.

Meta Analysis

Study name	Rate	Standard error	Variance	Lower limit	Upper limit	Z-Value	p-Value
Tan et al 2018	0.311	0.083	0.007	0.148	0.474	3.741	0.000
Smith et al 2014	0.310	0.067	0.004	0.179	0.441	4.625	0.000
Persson et al 2015	0.310	0.089	0.008	0.135	0.485	3.477	0.001
Flamiatos et al 2019	0.500	0.058	0.003	0.386	0.614	8.631	0.000
Ng et al 2010	0.240	0.048	0.002	0.146	0.334	4.996	0.000
	0.335	0.052	0.003	0.233	0.438	6.420	0.000

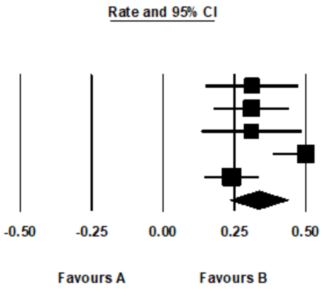

ORC no ERAS – Ileus rate.

Meta Analysis

Study name	Rate	Standard error	Variance	Lower limit	Upper limit	Z-Value	p-Value
Tan et al 2018	0.222	0.070	0.005	0.084	0.360	3.161	0.002
Koupparis_et_al-2015	0.060	0.033	0.001	-0.004	0.124	1.833	0.067
Smith et al 2014	0.080	0.034	0.001	0.013	0.147	2.349	0.019
Zhang et al 2020	0.112	0.021	0.000	0.071	0.153	5.375	0.000
Styn et al 2012	0.200	0.045	0.002	0.112	0.288	4.472	0.000
Kader et al 2013	0.180	0.042	0.002	0.097	0.263	4.243	0.000
	0.128	0.023	0.001	0.082	0.174	5.465	0.000

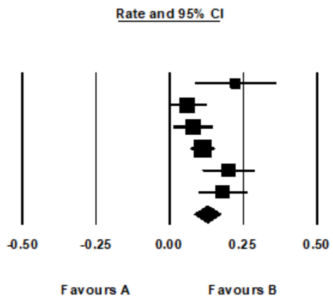

ORC no ERAS – Re-admission rate.

Meta Analysis

Study name	Rate	Standard error	Variance	Lower limit	Upper limit	Z-Value	p-Value
Bertolo et al 2018	0.130	0.047	0.002	0.039	0.221	2.793	0.005
Koupparis_et_al-2015	0.190	0.043	0.002	0.105	0.275	4.402	0.000
Chen et al 2019	0.210	0.038	0.001	0.135	0.285	5.480	0.000
Hussein et al 2020	0.090	0.014	0.000	0.063	0.117	6.614	0.000
	0.150	0.034	0.001	0.083	0.216	4.375	0.000

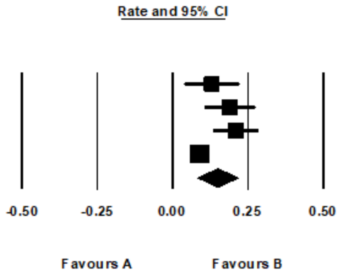

RARC ICUD ERAS – Transfusion rate.

Meta Analysis

Study name	Rate	Standard error	Variance	Lower limit	Upper limit	Z-Value	p-Value
	Statistics for each study						
Bertolo et al 2018	0.067	0.033	0.001	0.002	0.132	2.005	0.045
Collins et al 2015	0.190	0.038	0.001	0.116	0.264	5.065	0.000
Tan et al 2018	0.120	0.049	0.002	0.024	0.216	2.449	0.014
Koupparis_et_al-2015	0.080	0.028	0.001	0.025	0.135	2.857	0.004
Chen et al 2019	0.238	0.041	0.002	0.158	0.318	5.834	0.000
Tan et al 2019	0.197	0.058	0.003	0.084	0.310	3.409	0.001
Hussein et al 2020	0.210	0.021	0.000	0.169	0.251	10.102	0.000
	0.156	0.028	0.001	0.101	0.211	5.532	0.000

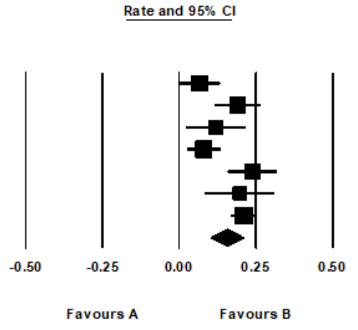

RARC ICUD ERAS – High grade complication rate.

Meta Analysis

Study name	Rate	Standard error	Variance	Lower limit	Upper limit	Z-Value	p-Value
	Statistics for each study						
Bertolo et al 2018	0.050	0.029	0.001	-0.007	0.107	1.732	0.083
Tan et al 2018	0.160	0.057	0.003	0.049	0.271	2.828	0.005
	0.073	0.026	0.001	0.022	0.123	2.828	0.005

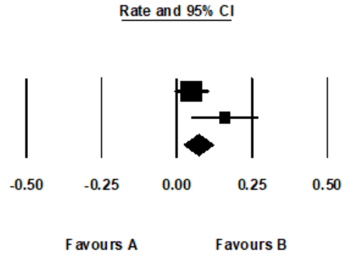

RARC ICUD ERAS – Ileus rate.

Meta Analysis

Study name	Rate	Standard error	Variance	Lower limit	Upper limit	Z-Value	p-Value
	Statistics for each study						
Bertolo et al 2018	0.150	0.050	0.003	0.052	0.248	3.000	0.003
Collins et al 2015	0.330	0.049	0.002	0.233	0.427	6.675	0.000
Tan et al 2018	0.120	0.049	0.002	0.024	0.216	2.449	0.014
Koupparis_et_al-2015	0.090	0.030	0.001	0.032	0.148	3.030	0.002
Chen et al 2019	0.364	0.050	0.003	0.265	0.463	7.215	0.000
Hussein et al 2020	0.270	0.024	0.001	0.224	0.316	11.455	0.000
	0.219	0.046	0.002	0.128	0.310	4.727	0.000

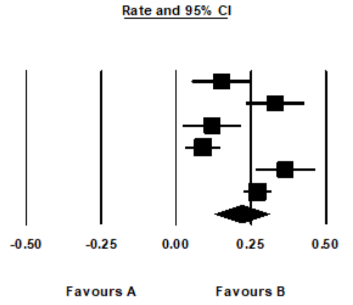

RARC ICUD ERAS – Re-admission rate.

Meta Analysis

Study name	Rate	Standard error	Variance	Lower limit	Upper limit	Z-Value	p-Value
Parekh et al 2013	0.400	0.141	0.020	0.123	0.677	2.828	0.005
Parekh et al 2018	0.240	0.040	0.002	0.162	0.318	6.000	0.000
Bertolo et al 2018	0.090	0.037	0.001	0.018	0.162	2.437	0.015
Hussein et al 2020	0.150	0.018	0.000	0.116	0.184	8.538	0.000
	0.173	0.038	0.001	0.099	0.246	4.582	0.000

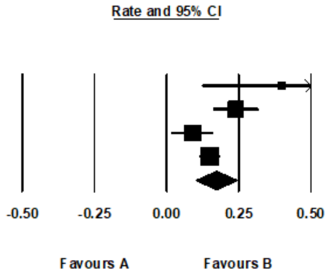

RARC ECUD ERAS – Transfusion rate.

Meta Analysis

Study name	Rate	Standard error	Variance	Lower limit	Upper limit	Z-Value	p-Value
Bochner et al 2015	0.220	0.061	0.004	0.101	0.339	3.633	0.000
Khan et al 2015	0.350	0.132	0.018	0.091	0.609	2.646	0.008
Nix et al 2009	0.150	0.087	0.008	-0.020	0.320	1.732	0.083
Parekh et al 2013	0.250	0.112	0.013	0.031	0.469	2.236	0.025
Parekh et al 2018	0.220	0.038	0.001	0.145	0.295	5.745	0.000
Bertolo et al 2018	0.060	0.032	0.001	-0.002	0.122	1.897	0.058
Saar_et_al-2013	0.096	0.056	0.003	-0.013	0.205	1.725	0.085
Tan et al 2019	0.191	0.053	0.003	0.087	0.295	3.604	0.000
Hussein et al BJUI 2020	0.240	0.022	0.000	0.196	0.284	10.800	0.000
	0.182	0.031	0.001	0.121	0.243	5.811	0.000

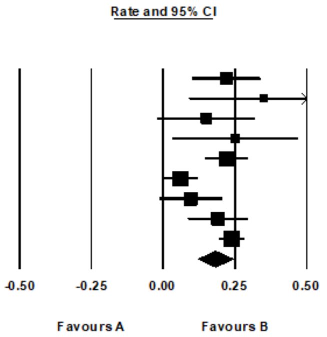

RARC ECUD ERAS – High grade complication rate.

Meta Analysis

Study name	Rate	Standard error	Variance	Lower limit	Upper limit	Z-Value	p-Value
Bochner et al 2015	0.230	0.062	0.004	0.109	0.351	3.715	0.000
Khan et al 2015	0.050	0.050	0.003	-0.048	0.148	1.000	0.317
Nix et al 2009	0.100	0.071	0.005	-0.039	0.239	1.414	0.157
Parekh et al 2018	0.220	0.038	0.001	0.145	0.295	5.745	0.000
Bertolo et al 2018	0.106	0.042	0.002	0.024	0.188	2.522	0.012
	0.143	0.037	0.001	0.070	0.215	3.874	0.000

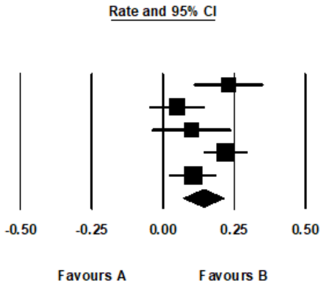

RARC ECUD ERAS – Ileus rate.

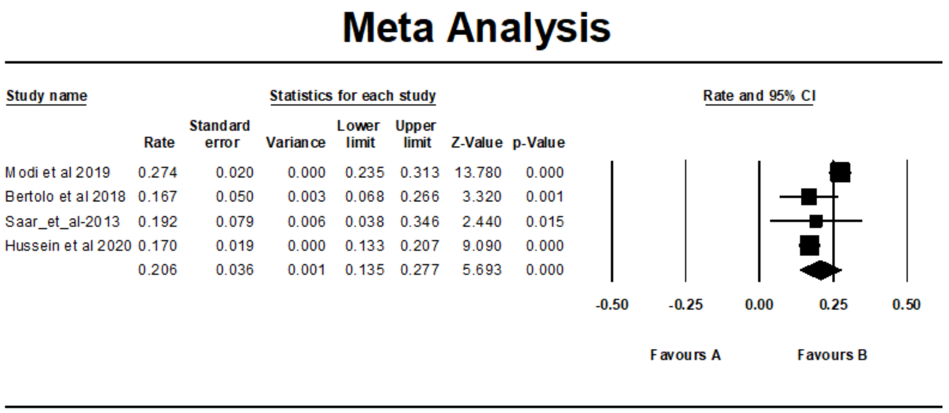

Meta Analysis

Study name	Rate	Standard error	Variance	Lower limit	Upper limit	Z-Value	p-Value
Modi et al 2019	0.274	0.020	0.000	0.235	0.313	13.780	0.000
Bertolo et al 2018	0.167	0.050	0.003	0.068	0.266	3.320	0.001
Saar_et_al-2013	0.192	0.079	0.006	0.038	0.346	2.440	0.015
Hussein et al 2020	0.170	0.019	0.000	0.133	0.207	9.090	0.000
	0.206	0.036	0.001	0.135	0.277	5.693	0.000

RARC ECUD ERAS – Re-admission rate.

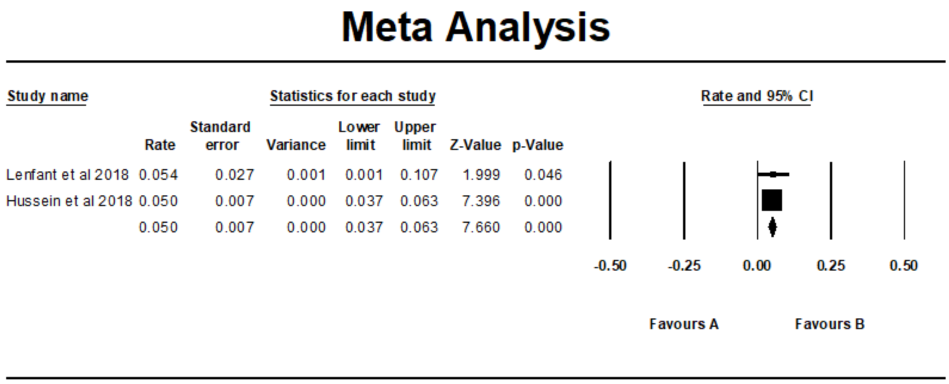

Meta Analysis

Study name	Rate	Standard error	Variance	Lower limit	Upper limit	Z-Value	p-Value
Lenfant et al 2018	0.054	0.027	0.001	0.001	0.107	1.999	0.046
Hussein et al 2018	0.050	0.007	0.000	0.037	0.063	7.396	0.000
	0.050	0.007	0.000	0.037	0.063	7.660	0.000

RARC ICUD no ERAS – Transfusion rate.

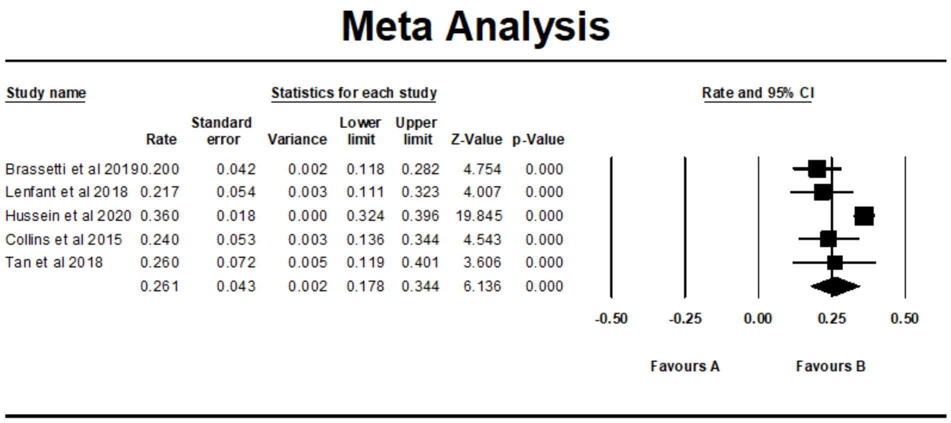

Meta Analysis

Study name	Rate	Standard error	Variance	Lower limit	Upper limit	Z-Value	p-Value
Brassetti et al 2019	0.200	0.042	0.002	0.118	0.282	4.754	0.000
Lenfant et al 2018	0.217	0.054	0.003	0.111	0.323	4.007	0.000
Hussein et al 2020	0.360	0.018	0.000	0.324	0.396	19.845	0.000
Collins et al 2015	0.240	0.053	0.003	0.136	0.344	4.543	0.000
Tan et al 2018	0.260	0.072	0.005	0.119	0.401	3.606	0.000
	0.261	0.043	0.002	0.178	0.344	6.136	0.000

RARC ICUD no ERAS – High grade complication rate.

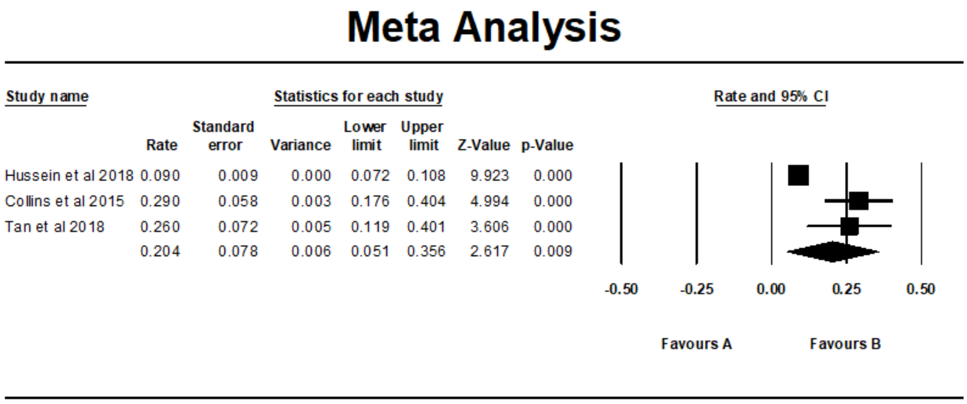

Meta Analysis

Study name	Rate	Standard error	Variance	Lower limit	Upper limit	Z-Value	p-Value
Hussein et al 2018	0.090	0.009	0.000	0.072	0.108	9.923	0.000
Collins et al 2015	0.290	0.058	0.003	0.176	0.404	4.994	0.000
Tan et al 2018	0.260	0.072	0.005	0.119	0.401	3.606	0.000
	0.204	0.078	0.006	0.051	0.356	2.617	0.009

RARC ICUD no ERAS – Re-admission rate.

Meta Analysis

Study name	Rate	Standard error	Variance	Lower limit	Upper limit	Z-Value	p-Value
Lenfant et al 2018	0.235	0.083	0.007	0.072	0.398	2.827	0.005
Hussein et al 2018	0.130	0.011	0.000	0.108	0.152	11.577	0.000
Flamiatos et al 2019	0.110	0.033	0.001	0.045	0.175	3.317	0.001
Styn et al 2012	0.020	0.020	0.000	-0.019	0.059	1.000	0.317
Kader et al 2013	0.150	0.039	0.002	0.074	0.226	3.873	0.000
	0.112	0.031	0.001	0.051	0.174	3.607	0.000

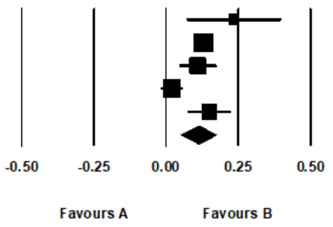

RARC ECUD no ERAS – Transfusion rate.

Meta Analysis

Study name	Rate	Standard error	Variance	Lower limit	Upper limit	Z-Value	p-Value
Lenfant et al 2018	0.222	0.081	0.007	0.064	0.380	2.747	0.006
Hussein et al 2018	0.230	0.015	0.000	0.201	0.259	15.399	0.000
Saar_et_al-2013	0.097	0.056	0.003	-0.013	0.207	1.734	0.083
Flamiatos et al 2019	0.100	0.032	0.001	0.038	0.162	3.162	0.002
Styn et al 2012	0.281	0.075	0.006	0.134	0.428	3.748	0.000
Ng et al 2010	0.169	0.045	0.002	0.081	0.257	3.745	0.000
Kader et al 2013	0.100	0.032	0.001	0.038	0.162	3.162	0.002
	0.164	0.031	0.001	0.102	0.225	5.219	0.000

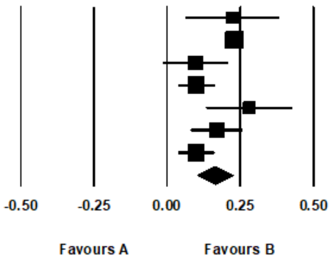

RARC ECUD no ERAS – High grade complication rate.

Meta Analysis

Study name	Rate	Standard error	Variance	Lower limit	Upper limit	Z-Value	p-Value
Flamiatos et al 2019	0.410	0.064	0.004	0.285	0.535	6.403	0.000
Ng et al 2010	0.160	0.044	0.002	0.074	0.246	3.644	0.000
	0.281	0.125	0.016	0.036	0.526	2.247	0.025

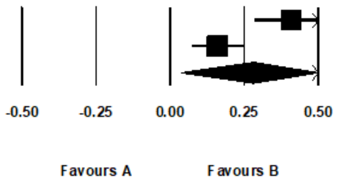

RARC ECUD no ERAS – Ileus rate.

Meta Analysis

Study name	Rate	Standard error	Variance	Lower limit	Upper limit	Z-Value	p-Value
Hussein et al 2018	0.240	0.015	0.000	0.210	0.270	15.730	0.000
Saar_et_al-2013	0.582	0.137	0.019	0.313	0.851	4.248	0.000
Styn et al 2012	0.840	0.130	0.017	0.586	1.094	6.481	0.000
Kader et al 2013	0.170	0.041	0.002	0.089	0.251	4.123	0.000
	0.393	0.086	0.007	0.225	0.561	4.578	0.000

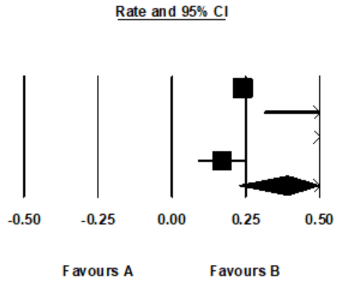

RARC ECUD no ERAS – Re-admission rate.

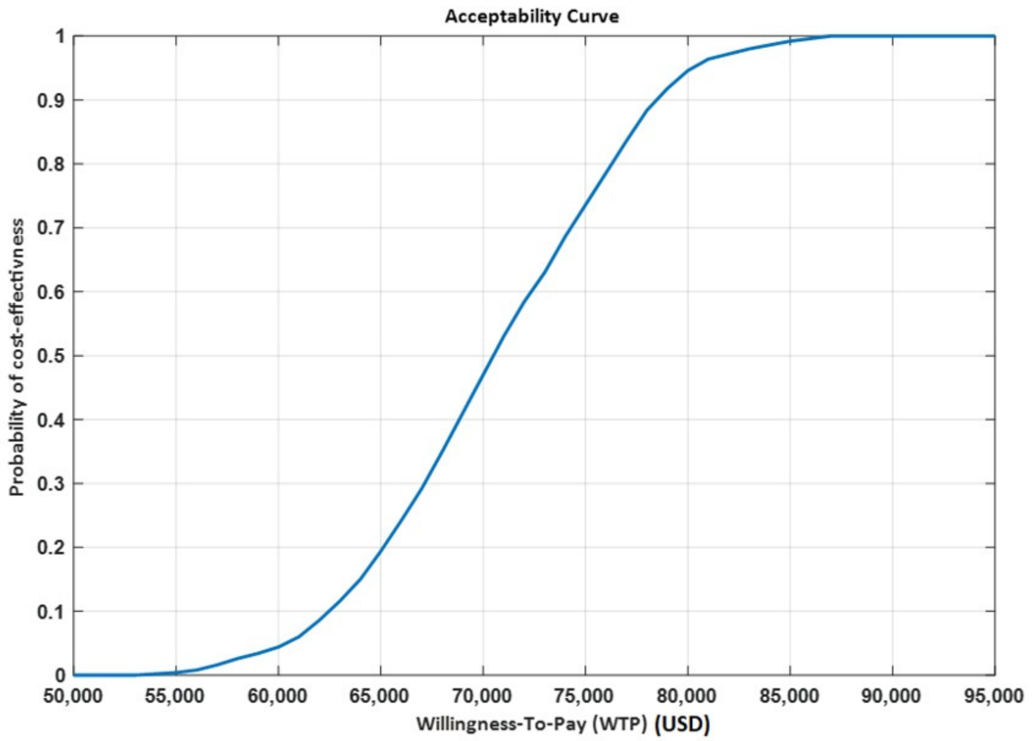

Acceptability curve in the US model. As CE is determined by the willingness to pay, this curve allows policy makers to determine the optimal WTP in each respective country.

References

1. Mmeje, C.O.; Martin, A.D.; Nunez-Nateras, R.; Parker, A.S.; Thiel, D.D.; Castle, E.P. Cost analysis of open radical cystectomy versus robot-assisted radical cystectomy. *Curr. Urol. Rep.* **2013**, *14*, 26–31. https://doi.org/10.1007/s11934-012-0292-7.
2. Zehnder, P.; Gill, I.S. Cost-effectiveness of open versus laparoscopic versus robotic-assisted laparoscopic cystectomy and urinary diversion. *Curr. Opin. Urol.* **2011**, *21*, 415–419. https://doi.org/10.1097/MOU.0b013e3283490582.
3. Smith, A.; Kurpad, R.; Lal, A.; Nielsen, M.; Wallen, E.M.; Pruthi, R.S. Cost analysis of robotic versus open radical cystectomy for bladder cancer. *J. Urol.* **2010**, *183*, 505–509. https://doi.org/10.1016/j.juro.2009.09.081.
4. Kulkarni, G.S.; Alibhai, S.M.; Finelli, A.; Fleshner, N.E.; Jewett, M.A.; Lopushinsky, S.R.; Bayoumi, A.M. Cost-effectiveness analysis of immediate radical cystectomy versus intravesical Bacillus Calmette-Guerin therapy for high-risk, high-grade (T1G3) bladder cancer. *Cancer* **2009**, *115*, 5450–5459. https://doi.org/10.1002/cncr.24634.
5. Stevenson, S.M.; Danzig, M.R.; Ghandour, R.A.; Deibert, C.M.; Decastro, G.J.; Benson, M.C.; McKiernan, J.M. Cost-effectiveness of neoadjuvant chemotherapy before radical cystectomy for muscle-invasive bladder cancer. *Urol. Oncol.* **2014**, *32*, 1172–1177. https://doi.org/10.1016/j.urolonc.2014.05.001.

6. Bansal, S.S.; Dogra, T.; Smith, P.W.; Amran, M.; Auluck, I.; Bhambra, M.; Sura, M.S.; Rowe, E.; Koupparis, A. Cost analysis of open radical cystectomy versus robot-assisted radical cystectomy. *BJU Int.* **2018**, *121*, 437–444. https://doi.org/10.1111/bju.14044.
7. Martin, A.D.; Nunez, R.N.; Castle, E.P. Robot-assisted radical cystectomy versus open radical cystectomy: A complete cost analysis. *Urology* **2011**, *77*, 621–625. https://doi.org/10.1016/j.urology.2010.07.502.
8. Wittig, K.; Ruel, N.; Barlog, J.; Crocitto, L.; Chan, K.; Lau, C.; Wilson, T.; Yuh, B. Critical Analysis of Hospital Readmission and Cost Burden After Robot-Assisted Radical Cystectomy. *J. Endourol.* **2016**, *30*, 83–91. https://doi.org/10.1089/end.2015.0438.
